# Transdisciplinary research in support of land and water management in China and Southeast Asia: evaluation of four research projects

**DOI:** 10.1007/s11625-016-0378-0

**Published:** 2016-06-08

**Authors:** Tuck Fatt Siew, Thomas Aenis, Joachim H. Spangenberg, Alexandra Nauditt, Petra Döll, Sina K. Frank, Lars Ribbe, Beatriz Rodriguez-Labajos, Christian Rumbaur, Josef Settele, Jue Wang

**Affiliations:** 1 Institute of Physical Geography, Goethe University Frankfurt, Altenhöferallee 1, 60438 Frankfurt am Main, Germany; 2 Albrecht Daniel Thaer-Institute of Agricultural and Horticultural Sciences, Humboldt-Universität zu Berlin, Luisenstrasse 53, 10099 Berlin, Germany; 3 Department of Community Ecology, UFZ-Helmholtz Centre for Environmental Research, 06120 Halle, Germany; 4 Sustainable Europe Research Institute Germany, Vorsterstrasse 97-99, Cologne, Germany; 5 Institute for Technology and Resources Management in the Tropics and Subtropics, Cologne University of Applied Sciences, 50679 Cologne, Germany; 6 Department of Geography, University of Cambridge, Downing Place, Cambridge, CB2 3EN UK; 7 Institute of Environmental Science and Technology, Autonomous University of Barcelona (ICTA-UAB), 08193 Cerdanyola del Vallès, Spain; 8 Chair of Hydrology and River Basin Management, Technical University Munich, 80333 Munich, Germany; 9 iDiv, German Centre for Integrative Biodiversity Research, Halle-Jena-Leipzig, Deutscher Platz 5e, 04103 Leipzig, Germany

**Keywords:** Evaluation, Interdisciplinarity, Knowledge co-production and integration, Land and water management, Sustainability problems, Transdisciplinarity

## Abstract

Transdisciplinary research (TDR) aims at identifying implementable solutions to difficult sustainability problems and at fostering social learning. It requires a well-managed collaboration among multidisciplinary scientists and multisectoral stakeholders. Performing TDR is challenging, particularly for foreign researchers working in countries with different institutional and socio-cultural conditions. There is a need to synthesize and share experience among researchers as well as practitioners regarding how TDR can be conducted under specific contexts. In this paper, we aim to evaluate and synthesize our unique experience in conducting TDR projects in Asia. We applied guiding principles of TDR to conduct a formative evaluation of four consortium projects on sustainable land and water management in China, the Philippines, and Vietnam. In all projects, local political conditions restricted the set of stakeholders that could be involved in the research processes. The set of involved stakeholders was also affected by the fact that stakeholders in most cases only participate if they belong to the personal network of the project leaders. Language barriers hampered effective communication between foreign researchers and stakeholders in all projects and thus knowledge integration. The TDR approach and its specific methods were adapted to respond to the specific cultural, social, and political conditions in the research areas, also with the aim to promote trust and interest of the stakeholders throughout the project. Additionally, various measures were implemented to promote collaboration among disciplinary scientists. Based on lessons learned, we provide specific recommendations for the design and implementation of TDR projects in particular in Asia.

## Introduction

Sustainable development requires the sustainable and integrated management of land and water. State-of-the art approaches for achieving such a management are Sustainable Land Management (World Bank 2006) and Integrated Water Resources Management (GWP 2000), which address both land and water albeit with a different focus. These concepts promote efficient use and combined management of land, water, and other natural resources as a pre-condition for optimal socio-economic development, while reducing negative anthropogenic impacts on the environment, for instance loss of biodiversity, soil degradation, water pollution, and water depletion that would undermine a sustainable development. Both approaches also promote knowledge sharing and generation among multidisciplinary scientists and stakeholders. Close collaboration among scientists from multiple disciplines is required to produce an interdisciplinary understanding of complex socio-ecological systems (Jury and Vaux 2005; Petts et al. 2006; Angelstam et al. 2013). Additionally, stakeholders from outside academia need to be involved to integrate their knowledge and to account for their diverse perspectives and interests in the variety of issues, including income generation, food security, gender relations, health, and environmental protection (Görg et al. 2014; Spangenberg et al. 2015b). “Stakeholders” are defined as those who are either (1) involved in the decision-making process, (2) affected by the decisions made, or (3) not involved in the decision-making process but important for a successful implementation of decisions made (Grimble and Wellard 1997; Reed et al. 2009). We regard “stakeholder” and “practitioner” as synonyms and use these terms interchangeably in this paper. Where not mentioned specifically, stakeholder is considered to be an institutional stakeholder (organization) which is represented by a key person (i.e., a stakeholder representative).

Bringing multidisciplinary scientists and multisectoral stakeholders together to address sustainability problems requires a transdisciplinary research approach. Transdisciplinary research (TDR) is a research mode that can be regarded as having progressed from disciplinary through multidisciplinary to interdisciplinary research with additional collaboration of multiple stakeholders from outside of academia (Pohl et al. 2008; Pohl 2010). TDR focuses on joint knowledge production and integration as well as mutual learning among scientists and stakeholders (CASS/ProClim 1997; Pohl and Hirsch Hadorn 2007; Jahn 2008; Stauffacher et al. 2008; Spangenberg 2011; Siew and Döll 2012; Scholz and Steiner 2015a). The types of knowledge to be integrated are system knowledge, target knowledge, and transformation knowledge (CASS/ProClim 1997). By integrating stakeholder knowledge with scientific knowledge, solutions that are developed based on system understanding while explicitly taking into account stakeholder values can likely be implemented.

Transdisciplinary approaches have been applied in various fields that deal with built and natural environments (Lawrence and Després 2004; Bergmann et al. 2012). These include land and water management (Scholz et al. 2000; Siew and Döll 2012; Schneider and Rist 2014; Zscheischler et al. 2014), urban studies (Ramadier 2004), regional planning and development (Stauffacher et al. 2008; Wiek and Walter 2009), sustainable agricultural development (Vandermeulen and van Huylenbroeck 2008), and conservation planning (Steventon 2008; Reyers et al. 2010). Application of TDR approaches has been increasing world-wide and is likely to increase further (Lang et al. 2012). Among the 104 transdisciplinary case studies reviewed by Brandt et al. (2013), the majority were conducted in Europe and North America by researchers located in the respective regions; others were carried out in Africa and Asia mainly by European researchers. According to Lang et al. (2012) and Brandt et al. (2013), the diverse experiences gained from TDR case studies across different countries, including a wide range of constraints and obstacles encountered (Scholz and Steiner 2015b), should be shared with the wider scientific community, particularly with those outside of the TDR community. This helps researchers and practitioners to understand better how TDR can be conducted in respective fields of application under specific socio-cultural contexts (Lang et al. 2012; Spangenberg 2011).

**Fig. 1:**
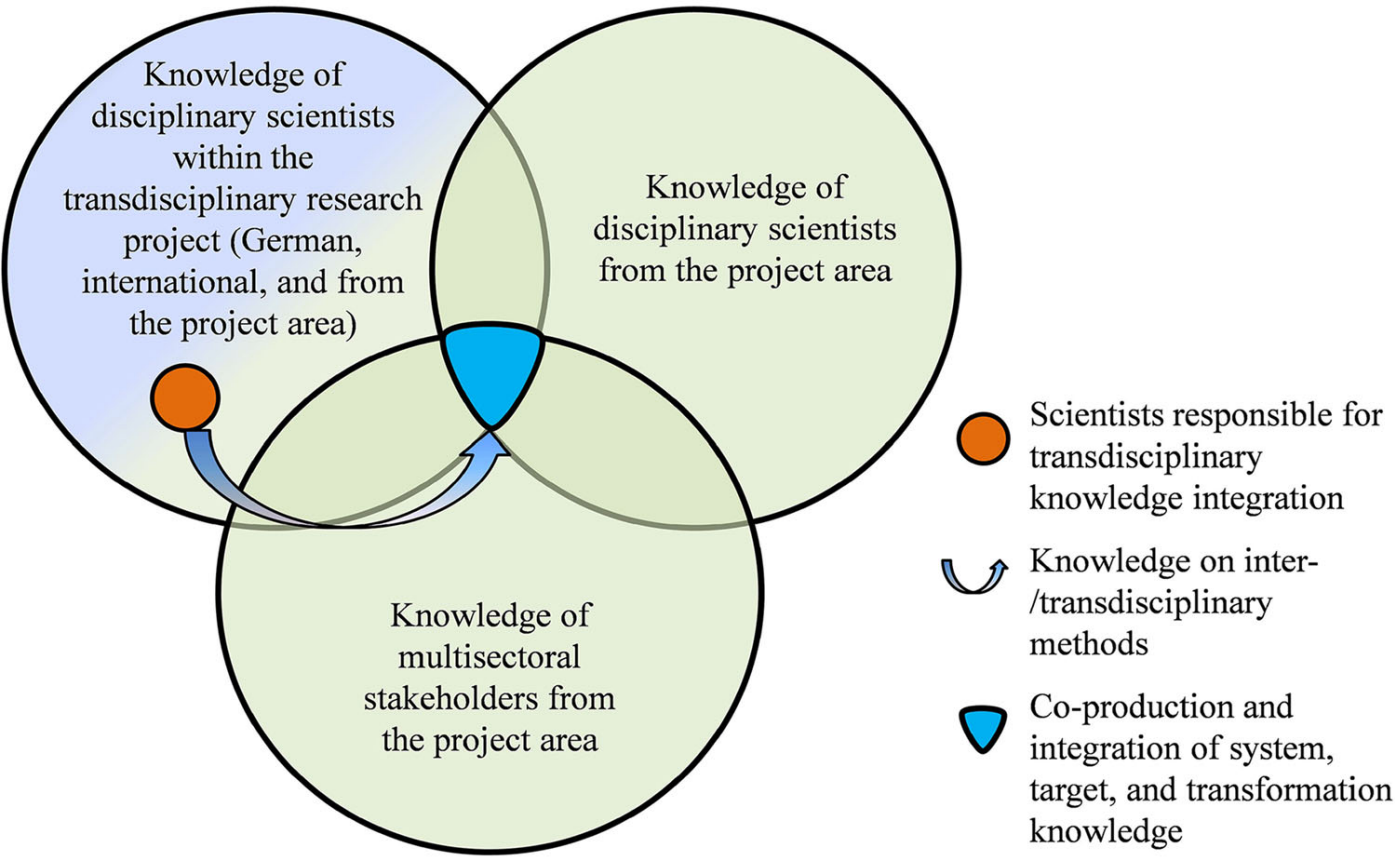
Knowledge co-production and integration among scientists within a transdisciplinary research project with knowledge of other scientists and stakeholders from the project area. Within each group, interdisciplinary and cross-sectoral knowledge integration, respectively, is facilitated by scientists responsible for knowledge integration using inter-/transdisciplinary methods. The *colour shades* of the *big circles* indicate integration of knowledge of scientists and stakeholders from the project area (*green*) and outside of the project area (*purple*). (Note: A number of scientists have interdisciplinary background)

In this paper, we synthesize experiences gained over a period of 4 years from four TDR projects in China, Vietnam, and the Philippines. In these projects, we focus on knowledge integration among multidisciplinary scientists within the respective projects with knowledge of multidisciplinary scientists and multisectoral stakeholders from the respective project areas (Fig. 1) as well as organizational issues of the TDR projects. Using the guiding questions developed by Lang et al. (2012), we evaluate whether TDR was really performed in these projects. Based on this evaluation, we then make recommendations on how TDR can be done better. Our ultimate goal is to share unique experiences and lessons learned with researchers who are interested in conducting TDR in foreign countries, particularly in Asia, as well as with Asian researchers who are keen to collaborate with foreign researchers to bring TDR projects to fruition. Evaluation of project outcomes and analysis of the link between the TDR process or project features and the project outcomes, including social learning, is beyond the scope of this paper.

Our regional projects deal with land and water management under land use and climate change and put a focus on the analysis and management of ecosystems and their services. In the next section, we describe the four projects. We then present the guiding questions used for evaluating the projects. Subsequently, the evaluation results are discussed, including the challenges encountered over the course of the projects and ways to adapt TDR. The conclusions include recommendations for conducting TDR in Asian countries like China, Vietnam, and the Philippines.

## Description of transdisciplinary research projects

**Fig. 2:**
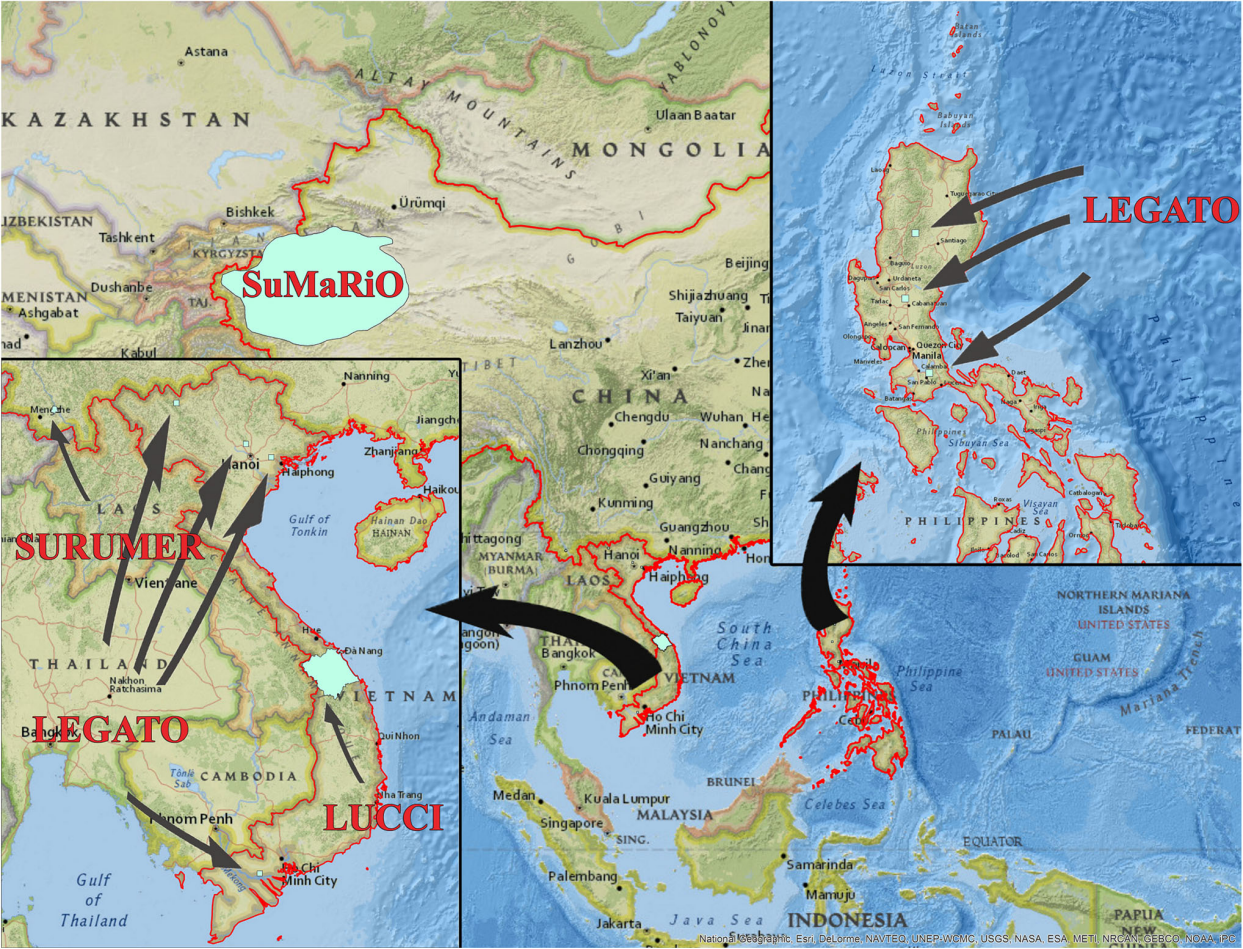
Transdisciplinary research projects conducted in China (SuMaRiO, SURUMER), Vietnam (LEGATO, LUCCi), and the Philippines (LEGATO)

The regional distribution of the four TDR projects in China, Vietnam, and the Philippines is shown in Fig. 2. The projects are among 12 regional projects funded by the German Federal Ministry of Education and Research (BMBF) under the research program “Sustainable Land Management Module A” (http://www.fona.de/en/10073). All projects started in 2010 or 2011 and are funded for a total of 5 years. Other regional projects are located in Russia, Africa, and the Baltic region. The funding measure aims at generating scientific knowledge for an improved understanding of sustainable land and water management and at providing relevant strategies for action in the study areas, including suitable technologies and integrated solutions. All of the 12 regional projects are supported by the “bridge-project” GLUES (Global Assessment of Land Use Dynamics, Greenhouse Gas Emissions and Ecosystem Services) that facilitates synthesis, data sharing, and knowledge exchange among the projects. The goals, foci, targeted outputs, and the scales of the four regional projects are listed in Table 1. Each project differs in the comprehensiveness of the problem fields addressed.

**Table 1:** Overview of four transdisciplinary research projects in China, Vietnam, and the Philippines

	1 SuMaRiO^a^	2 SURUMER^b^	3 LEGATO^c^	4 LUCCi^d^
Project region	Northwest China (Xinjiang Uyghur Autonomous Region)	South China (Yunnan Province)	North and South Vietnam, Luzon Island in the Philippines	Central Vietnam
Goal	To support oasis management along the Tarim River under conditions of climatic and societal changes	To develop an integrative, applicable, and stakeholder-validated concept for sustainable rubber cultivation in southern Yunnan	To develop concepts of landscape scale management and ecological engineering practices, contributing to the sustainable development of irrigated rice cultivation in Southeast Asia	To provide a scientific basis for the development of sustainable land use and water management strategies considering socio-economic development, population growth, and impacts of climate change on land and water resources
Project focus	Phase 1: Analysis of streamflow under climate change, water demand and biomass production, ecosystem functions and services, and socio-economic assessment Phase 2: Implementation of research results Throughout the project: stakeholder dialogue focused on joint problem definition, participatory scenario development, and identification of implementable strategies, considering ecosystem services	Phase 1: Situational analysis of ecosystem functions and services (multidisciplinary focus)	Preparation phase: Identification of relevant issues and experimental sites jointly with stakeholders	Preparation phase: Identification of relevant issues with stakeholders
				After project start: Integrated modelling and scenario development (regional climate change scenarios, GHG emission estimates and carbon stock changes; flood, drought and salt water intrusion; distribution of biodiversity patterns; impact of land use changes on water resources). Based on modelling results and scenarios land use planning and water management strategies are developed and implemented. Stakeholders were involved in data collection and scenarios and strategies development
			Phase 1: Intensive communication, stakeholder discourses for co-generation of target knowledge, adaptation of research questions, data gathering	
		Phase 2: Integration of findings into new land use concepts (interdisciplinary focus)		
		Phase 3: Transfer of scientific concept into practical land use and policies.	Phase 2: Disciplinary-based information distillation, processing and evaluation; communication of results with stakeholders	
		Throughout the project: stakeholder discourses focused on mutual situational analysis, participatory scenario development, and discussion of trade-offs		
			Phase 3: Application, dissemination and implementation	
Targeted output	Improved knowledge about the relation between water allocation and ecosystem services, and on impact of climate change on water resources; concepts and recommendations; a decision support tool for supporting land and water management that takes ecosystem services into account	Improved management concepts, land use policies, strategies, measures	Development of sustained use of landscape scale management and ecological engineering (EE) concepts, demonstration of EE benefits leading to further diffusion of EE practices and co-generated knowledge, enhanced informal lower level exchange	Concepts and strategies for sustainable land and water management available for scientists and decision makers in form of Integrated Modeling and Decision Support System (DSS VGTB), River Basin Information System (RBIS), and River Basin Information Center (RBIC)
Number of collaborating universities and research institutes within the project	19 (11 in Germany, 8 in China)	18 (9 in Germany, 9 in China)	22 (11 in Germany, 2 in the Philippines, 4 in Vietnam, 1 in Spain, 1 in United Kingdom, 1 in Bulgaria, 2 international organisations)	14 (6 in Germany, 5 in Vietnam, 3 international organization
Number of institutional stakeholders from the project area involved (those not within the project). I: universities and research institutes; O: organizations from outside academia	I: 7	I: 3	I: 15	I: 5
				O: 21
			O: 10	
		O: approx. 20 (changing over time)		
	O: 8			

^a^Sustainable management of river oases along the Tarim River, Northwest China (http://www.sumario.de; Rumbaur et al. 2015)

^b^Sustainable rubber cultivation in the Mekong Region—development of an integrative land-use concept in Yunnan Province, Southwest China (https://surumer.uni-hohenheim.de/)

^c^Land-use intensity and ecological engineering—assessment tools for risks and opportunities in irrigated rice based production systems, Vietnam and the Philippines (http://www.legato-project.net; Settele et al. 2013)

^d^Land-use and climate change interactions in the Vu Gia Thu Bon River Basin, Vietnam (http://www.lucci-vietnam.info)

### Regional project 1: SuMaRiO

“**Su**stainable **Ma**nagement of **Ri**ver **O**ases along the Tarim River” (SuMaRiO) is a German-Chinese collaboration project funded since March 2011 (Rumbaur et al. 2015). The Tarim River Basin is located in the southern part of Xinjiang Uyghur Autonomous Region, Northwest China. It is the largest inland basin in China with approximately one million km^2^ and is inhabited by about eight million people. Due to the arid climate, water resources for the basin stem almost exclusively from high mountain glaciers and snow melt that is transported to water users by a small number of rivers. Water allocation and the impact of water use in different regions (upstream, midstream, downstream) by different users (including irrigated crops, natural riparian vegetation, and irrigated urban and per-urban vegetation) on the environment (e.g., soil salinization, degradation of riparian vegetation) are the major issues of concern in the region (Shen and Lein 2005; Thevs et al. 2015). The Tarim Basin Water Resources Commission seeks an improved land and water management, particularly with regard to water allocation and use. The commission is a basin-level water management body that comprises governmental organizations from different sectors (including water, agriculture, and forestry) and administrative levels (provincial, prefecture, and county).

### Regional project 2: SURUMER

“**Su**stainable **RU**bber cultivation in the **Me**kong **R**egion” (SURUMER) is a German-Chinese collaboration project funded since December 2011 (https://surumer.uni-hohenheim.de/). The project is implemented in Xishuangbanna in the southern part of the Chinese Yunnan province, which was once covered by tropical rainforests. The area was converted to large-scale rubber plantations and has become the second largest rubber growing region in China. Rubber production provides a high income potential to local farmers. However, switching from traditional, subsistence-oriented farming to intensive rubber cultivation has degraded the natural forest ecosystem and its services, with a loss of plant and animal biodiversity as well as clean water. An integrative land use concept is required to foster socio-economic development, while protecting the environment.

### Regional project 3: LEGATO

“**L**and use intensity and **E**cological en**G**ineering—**A**ssessment **T**ools for risks and **O**pportunities in irrigated rice-based production systems” (LEGATO), funded since March 2011, covers seven study areas in the Philippines (Luzon Island) and in Vietnam, and seeks practical options for sustainable rice cultivation in both mountainous regions and lowlands (Settele et al. 2013; Klotzbücher et al. 2015; Schmidt et al. 2015; Westphal et al. 2015). The mountain regions of the Philippines and Vietnam are inhabited by ethnic minorities with strong roots in animist belief systems. They show more similarities between them, in some respects, than with the rest of their respective countries. Filipino farmers own their land and trade with it (restricted by tradition), which is more important for small and medium size farmers who produce for local markets. Vietnamese farmers have a land use right, while the ground remains state property. The structure and intensity of land use vary widely, among areas and between countries. Subsistence agriculture prevails in the mountainous areas. In the Philippines, farmers use traditional rice varieties without chemical inputs, while in Vietnam high yielding varieties using chemical input are widespread. Low income of farmers and loss of traditional knowledge of rice cultivation, including terrace management, are main concerns. Regionally, tourism provides additional income but both benefit sharing and integration into the rice cycle of activities are prone to problems. In both countries’ lowlands, agricultural sustainability problems involve medium to high levels of external inputs, insufficient agricultural extension as compared to chemical companies’ influence, rising costs, low farm worker income, and significant levels of water pollution. The impact of insecticide use to control planthoppers is a major issue. Knowledge on *timely* insecticide spraying is required to avoid killing useful biocontrol agents (predators and parasitoids) (Heong 2009; Spangenberg et al. 2015a).

### Regional project 4: LUCCi

The project “**L**and-**U**se and **C**limate **C**hange **i**nteractions in the Vu Gia-Thu Bon River Basin, Vietnam” (LUCCi) has been completed by the time of this evaluation. The project started in July 2010 after it had been designed together with the local partners since early 2009. One of the major issues in the Vu Gia-Thu Bon basin is upstream hydropower development that modifies downstream hydrological conditions, leading to decreasing water availability for irrigation during the dry season and salt water intrusion into rice irrigation channels in the coastal areas. Taking into account national and regional land and water use planning strategies, recent development trends, regional climate projections, hydropower development as well as potential greenhouse gas emissions from agricultural and other land uses, the project aims at providing a scientific basis to develop optimized land use and water resources management strategies for Central Vietnam. On the one hand, greenhouse gas emissions from different land uses and land covers are quantified. On the other hand, possible climate change impacts on existing land uses are analyzed and suitable adaptation strategies are developed (http://www.lucci-vietnam.info).

## Methods

To systematically evaluate the four TDR projects in China, Vietnam, and the Philippines, we used the guiding questions developed by Lang et al. (2012) (Table 2). “We” is referred to as the authors of this paper who are directly involved in designing, implementing, and evaluating the respective TDR projects. We reflected on issues related to the three main phases of a TDR, including organization of a TDR project and knowledge integration, by answering the guiding questions. Feedbacks gathered from other researchers within and outside of the respective consortiums as well as stakeholders in the respective countries were taken into account in this evaluation. All information was compiled, analyzed descriptively, and organized according to the TDR phases encompassing the guiding questions. Only those projects funded by BMBF under the research program “Sustainable Land Management Module A” and located in Asian region were selected for evaluation. These projects address complex sustainable land and water management problems which require collaborative efforts between researchers and stakeholders to develop knowledge-based solutions.

**Table 2:** Transdisciplinary research phases and the pertaining questions for guiding evaluation (modified from Lang et al. 2012)

Transdisciplinary research phase	Guiding question
Phase A preparation	
A.1 Build a collaborative research team (scientists + stakeholders)	Does the project team include all relevant expertise, experience, and other relevant ‘‘stakes’’ needed to tackle the sustainability problem in a way that provides solution options and contributes to the related scientific body of knowledge?
A.2 Create joint understanding and define the sustainability problem to be addressed	Does the project team reach a common understanding of the sustainability problem to be addressed and does the team accept a joint definition of the problem?
A.3 Collaboratively define the boundary/research object, research objectives as well as specific research questions, and success criteria	Is a common research object or guiding question, with subsequent specified research objects and questions, formulated, and do the partners agree on common success criteria?
A.4 Design a methodological framework for collaborative knowledge production and integration	Does the project team agree upon a jointly developed methodological framework that defines how the research target will be pursued in Phase B and what transdisciplinary settings will be employed? Does the framework adequately account for both the collaboration among the scientific fields and with the practice partners?
Phase B research	
B.1 Assign and support appropriate roles for practitioners and researchers	Are the tasks and roles of the actors from science and practice involved in the research process clearly defined?
B.2 Apply and adjust integrative research methods and transdisciplinary settings for knowledge generation and integration	Does the research team employ or develop methods suitable to generate solution options for the problem addressed? Does the team employ or develop suitable settings for inter- and transdisciplinary cooperation and knowledge integration?
Phase C application	
C.1 Realize two-dimensional integration	Are the project results implemented to resolve or mitigate the problem addressed? Are the results integrated into the existing scientific body of knowledge for transfer and scaling-up efforts?
C.2 Generate targeted products for both parties	Does the research team provide practice partners and scientists with products, publications, services, etc. in an appropriate form and language?
C.3 Evaluate scientific and societal impact	Are the goals being achieved? What additional (unanticipated) positive effects are being accomplished?
Cutting across the three phases	
D.1 Facilitate continuous formative evaluation	Is a formative evaluation being conducted involving relevant experts related to the topical field and transdisciplinary research (throughout the project)?
D.2 Mitigate conflict constellations	Do the researchers/practitioners prepare for/anticipate conflict at the outset, and are procedures/processes being adopted for managing conflict as and when it arises?
D.3 Enhance capabilities for and interest in participation	Is adequate attention being paid to the (material and intellectual) capabilities that are required for effective and sustained participation in the project over time?

The TDR phases that encompass the guiding questions are A: Building a collaborative research team, collaborative problem framing, and design of methodological framework; B: Co-creation of solution-oriented and transferable knowledge through collaborative research; and C: (Re-)integrating and applying the co-created knowledge. Phase A can be considered as a preparation phase, Phase B as research phase, and Phase C as application phase (Lang et al. 2012). According to Lang et al. (2012), the set of guiding questions can be used to conduct different types of evaluation, namely ex-ante evaluation, formative evaluation (i.e., during the research process), or ex-post evaluation. In this paper, we focus on formative evaluation of Phase A and B.

## Evaluation results

Results of the evaluation of the four TDR projects are presented following the sequence of guiding questions listed in Table 2. Evaluation of Phase C is not included because all projects except LUCCi are ongoing.

### Phase A: preparation

#### A. 1 Build a collaborative research team

The four regional projects were initiated by German researchers and research partners from the respective countries in Asia (Table 1). In LEGATO project, researchers from other European countries are also involved. The research teams of the respective projects, which are composed of 14 to 22 research institutions (Table 1), are dominated by natural scientists. All projects were, in fact, initiated by natural scientists who asked social scientists to join to fulfil the requirements of the funding call for TDR. Each of the research team includes researchers with different degrees of experience in the field of investigation. While PhD students and post-doctoral researchers have less academic experience than principal investigators, some of the doctoral students and post-doctoral researchers have a better understanding about the socio-cultural differences and the ways of communication in the respective countries. This knowledge is essential for a successful (transdisciplinary) research in a foreign country (van den Hoek et al. 2012).

In each project, staff changes occurred during the project period as some researchers left the projects due to various reasons. Most staff members were financed for less than the project duration of 5 years, in particular the doctoral students that were typically financed for only 3 years. In the case of SURUMER, the German principle researcher team at the beginning of the project was already different from the one that applied for the project. Although researchers could be replaced by new ones, the progress of the project was certainly affected as time was needed to get familiarize with the project.

Building a collaborative research team that comprised all relevant stakeholders during the preparation phase could not be realized in all regional projects. Instead, the stakeholders were involved at different stages of the research processes to different degrees. Stakeholders include representatives from different organizations and sectors such as governmental, non-governmental, and private sectors as well as individual local people (Table 3). The LEGATO team could already integrate stakeholders during a funded 6-month project preparation period because the project coordinator has long-term experience in the project areas and relevant background knowledge about the problem at hand. During this period, relevant experts and stakeholders from local and provincial levels were consulted to shape the final application for project funding (Settele et al. 2013). In the LUCCi project, an interdisciplinary research team consisting of German and Vietnamese researchers was defined and the stakeholders were also involved during the preparation of project proposal, although the project did not get additionally funding for the preparation period. The stakeholders provided information on local problems and formulated research questions together with the research team. By so doing, the project objectives could be defined considering the stakeholder needs and research demand in the project area during the preparation stage. The local partners (nature reserve bureau) of the SURUMER project were involved in the discussion about the concept of the overall project immediately after the project had been kicked off; throughout the project period the intensity of interactions between stakeholders and subproject teams varied. In SuMaRiO, the key stakeholders from the river basin organization and the provincial water resources bureau became involved only after the project start, too. In all projects, some of the relevant stakeholders might not have been engaged in the transdisciplinary processes to the degree desirable because they were involved at a later stage.

**Table 3:** Category of stakeholders involved in the four transdisciplinary research projects in China, Vietnam, and the Philippines

Types of stakeholders	SuMaRiO	SURUMER	LEGATO	LUCCi
Government	Yes	Yes	Yes	Yes
Private sector	No	Yes	Yes	Yes
Non-governmental organizations	No	Yes	Yes	No
International organizations	No	Yes	Yes	Yes
Individuals (e.g., farmers, households, residents)	Yes	Yes	Yes	Yes
Academia	Yes	Yes	Yes	Yes

The early involvement of relevant stakeholders is important for creating the ownership of the respective projects by stakeholders from the beginning of the project as well as for ensuring successful implementation of research results during the results application phase. However, getting the right stakeholders to involve at the right time in the TDR process of each regional project was generally challenging due to several reasons. Due to strong political constraints in the Xinjiang Uyghur Autonomous Region that suffers from ethnic tensions and violent conflicts, it was not possible to involve non-governmental or private organizations in SuMaRiO. Most governmental stakeholders were reluctant to be involved officially because according to them the project was not officially endorsed by the Chinese central government (Siew et al. 2014).

In both Chinese projects, SuMaRiO and SURUMER, one of the Chinese research partners selected the representatives of stakeholders that were invited to participate in the stakeholder dialogue from an informal network of people personally known to them. In both cases, these research partners were high-ranking and influential. In SURUMER, governmental stakeholders tended to send higher ranking representatives because the local partner was represented by its director. Involvement of stakeholders from county and prefecture levels was easier than of those from the provincial level. In the case of LEGATO (the Philippines and Vietnam) and LUCCi (Vietnam), a certain level of association with governmental stakeholders at the local, provincial and national levels and farmers was necessary. In Vietnam, farmers could not be selected freely by the researchers; the selection must be agreed first by the administration officials. According to the officials, local administrative structures and hierarchies must be considered when identifying stakeholders, while the freedom of choice of stakeholders was constrained by the local political framework. Due to these restrictions, relevant stakeholders have been excluded which may lead to insufficient consideration of diverse interests during the process of addressing conflicting objectives (trade-offs) that arise from (competing) resource use (Grimble and Wellard 1997).

Some stakeholder representatives might have had interest in participation in the respective transdisciplinary processes, but did not have the capacity to do so in a meaningful way. For instance, stakeholder representatives from the government sector usually have limited time to participate in a workshop and even less so to continuously participate in a series of workshops. In SuMaRiO, the government staff could only allocate about three to 4 hours of their time for each workshop. When receiving last minute tasks from the government, they had to cancel their participation. In general, research activities in TDR are given lower priority, particularly when the project is not endorsed by the government (Siew et al. 2014). In LUCCi, there was a lack of financial means for stakeholders to become involved actively. In Vietnam, as well as in China, stakeholder and farmer participation requires financial compensation. Additional budget would have been needed, but getting extra funding for this purpose was not possible. The LEGATO team did not face this problem because the budget needed was already included in the financial plan.

#### A. 2 Create joint understanding and define the sustainability problem to be addressed

The research team of each regional project reached a common understanding of the sustainability problem to be addressed and developed a joint problem definition together with the stakeholders involved in the processes at different stages. In LEGATO and LUCCi, the sustainability problem was discussed and identified jointly with stakeholders already during the preparation phase (comp. A.1). The core team of SURUMER used the outcomes of a previous German-Chinese collaboration project “Living Landscape China” (https://lilac.uni-hohenheim.de/en/index.php) as a basis for discussion with stakeholders to achieve a common understanding after the project start. In SuMaRiO, a problem perception shared by researchers and stakeholders was achieved at the second stakeholder workshop.

#### A. 3 Collaboratively define the boundary/research object, research objectives as well as specific research questions, and success criteria

German and non-German researchers of all four regional projects (mainly the principal investigators of subprojects) were involved in writing proposals according to the requirements set by the call of the funding agency BMBF. They defined the overall boundary/research object, research objectives, and research questions collaboratively. The cross-cutting question was how ecosystem services can be sustained and used to support livelihoods by means of improved land and water management under climate, land use, and societal change. Only in LEGATO and LUCCi, stakeholders directly influenced the definition of research object and objectives, as only in case of these projects they were consulted by the research teams during project preparation. However, Chinese scientists involved in defining research object and objectives in SuMaRiO and SURUMER often are in close contact to governmental stakeholders and may have reflected stakeholder perceptions.

Common success criteria were neither defined nor agreed on by the research teams and stakeholders of the respective projects. They were believed to be either too vague to be meaningful, or—if more specific—could hardly fit to the diverse types of research done within individual subprojects.

#### A. 4 Design a methodological framework for collaborative knowledge production and integration

Table 4 shows diverse approaches and methods for stakeholder involvement and collaborative knowledge production and integration in the respective regional projects. The approaches and methods were designed and selected by the team responsible for the execution of the TDR process in each project during proposal writing stage. They were chosen based on the objectives of the respective projects as well as the preferences and expertise of the researchers. The approaches for stakeholder involvement were stakeholder dialogues and stakeholder discourses. Knowledge integration was supported by inter alia integrated modelling and assessment methods and (participatory) scenario development. In LEGATO, state-of-the-art participatory methods like integrative iterative discourses and citizen science were also applied for knowledge integration. All the approaches and methods were designed and selected in light of their ability to facilitate collaborative knowledge production and integration. Their suitability and adequacy in the actual situations was tested while applying them in the TDR process.

**Table 4:** Transdisciplinary approaches and methods designed and selected for stakeholder involvement and knowledge production and integration in the four case studies in China, Vietnam, and the Philippines

	SuMaRiO	SURUMER	LEGATO	LUCCi
Stakeholder involvement approach	Stakeholder dialogue (Interviews, workshops)	Stakeholder discourses (informal and formal interviews, workshops)	Stakeholder discourses (interviews, focus group discussion, direct or indirect participant observation) with feedback rounds, workshops, conferences, publications	Stakeholder dialogue (workshops, roundtable discussion, interviews regarding scenario development)
Methods of knowledge integration	Actor modelling, Bayesian network modelling, participatory scenario development, decision support system	Integrated modelling, participatory scenario development	Integrated assessment, integrative iterative discourses, scenario development, monitoring, direct collaboration in citizens science	Integrated modelling, participatory scenario and strategy development

### Phase B: research

#### B. 1 Assign and support appropriate roles for practitioners and researchers

The roles of German and non-German researchers in the respective regional projects were defined according to the tasks they were involved in. Disciplinary researchers in each subproject were responsible for carrying out field experiments, interviews, or modelling in the respective fields of study. German researchers responsible for enabling TDR identified and engaged stakeholders in the research processes together with their respective project partners from the project areas. Additionally, they integrated research results gained from different subprojects/disciplines with knowledge of diverse stakeholders from inside and outside academia. In SuMaRiO, the main stakeholder dialogue on overall land and water management along the Tarim was complemented by a second stakeholder dialogue on the sub-theme, dust and heat stress mitigation by urban and peri-urban vegetation (Frank et al. 2014). In addition, many of the disciplinary scientists organized workshops about their specific research in which both Chinese researchers and stakeholder representatives took part (in total 21 workshops between March 2011 and July 2015 in SuMaRiO).

The overall coordination, monitoring of project milestones, project reporting, and strategic decision making in each project were facilitated by a German project coordinator, who is a researcher as well. While a researcher assumed multiple roles, the roles of the coordinators and researchers responsible for TDR overlapped sometimes, for example with regard to leading the production of scientific outputs within the respective projects. To improve the overall project integration and coordination, SURUMER built a “project monitoring and strategy team” which proved to be quite effective. The steering group consisted of five members, including representatives of different important subgroups. The members were the project leader, the coordinator, one representative of natural sciences, one of social sciences, and one of modelling.

In all projects, stakeholders from inside and outside academia, who were involved as interview partners, participants in workshops, discussion groups, or citizen science, were co-creators of knowledge as well as informants who provided important knowledge and insights on the relevant issues in the study regions. In LEGATO project for example, terrace rice farmers, agricultural advisors, administrators, and tourism operators identified practical options for maintaining rice cultivation in the uplands and taking best advantage of the terraces to improve farmers’ livelihoods, combining sustainable agriculture and eco-tourism.

#### B. 2 Apply and adjust integrative research methods and transdisciplinary settings for knowledge generation and integration

Transdisciplinary settings and methods designed in Phase A.4 were applied and adjusted according to the socio-cultural contexts in the study areas to achieve optimal knowledge generation and integration. In SuMaRiO, the actor modelling method (Titz and Döll 2009), which was planned to be used for integrating the problem perceptions of institutional stakeholders from outside academia in the form of a perception graph, was modified by first eliciting the problem perceptions of Chinese scientists (Siew et al. 2014). As described in A.1, the research team of the SuMaRiO project had limited access to stakeholders, while governmental stakeholders were reluctant to be interviewed officially. At workshops, a combination of methods was applied to facilitate communication as well as to elicit knowledge of stakeholders and scientists. For instance, the World Café format was used at the first workshop for encouraging discussions in smaller groups. Short questionnaires were filled out by the participants during the workshops to collect specific information regarding the problems faced in different sections of the Tarim River (upstream, midstream, and downstream). Questionnaires are a useful means because some participants did not express their views openly in plenary or even small group discussion sessions likely due to hierarchy issues. At workshops, participants who ranked lower in the administrative hierarchy usually showed respect to those higher in ranking (most often directly related to the age of the person). Gaining direct input from stakeholders, particularly those from the governmental organizations, was almost impossible in SuMaRiO. Stakeholders preferred to provide feedback on input given by researchers, for instance with regard to the development of two qualitative scenarios and possible management measures. While this type of stakeholder intervention maybe considered rather passive, it was found to be both effective and efficient in light of the limited time available for generating robust results at the workshops. Nevertheless, a continuous knowledge development and integration with stakeholders was impossible in SuMaRiO, as there was a very large fluctuation of participants from workshop to workshop. The other projects did not face this problem.

In SURUMER, the methodology of interaction was shaped by the SURUMER project team after internal and external evaluation towards participatory problem analysis and scenario development. According to stakeholders, they preferred to discuss preliminary research results and possible management options for sustainable rubber cultivation in a concrete way instead of problems and scenarios they perceived to be too abstract to be of interest. Based on stakeholder feedbacks, the SURUMER team had strengthened the discussion about concrete results and options for sustainable rubber cultivation (Aenis and Wang 2014).

The approaches and methods applied in LUCCi and LEGATO projects for knowledge generation and integration did not require major adaptation. In the LUCCi project, workshops, working meetings of small research groups, visits to the relevant institutions as well as interviews and questionnaires were applied as transdisciplinary settings for knowledge integration. All project activities, including data collection as well as scenario and strategies development were carried out jointly by the Vietnamese and German researchers in strong collaboration with the local stakeholders from public and private sector. Identified land use planning and water management strategies were implemented in the final phase of the project. As compared to other projects, LEGATO focused stronger on integrating knowledge of local farmers, particularly regarding farming practices. Other projects focused more on integrating system, target, and transformation knowledge of institutional stakeholders. In LEGATO, the joint preparation phase was followed by intensive communication, stakeholder discourses and as a result of this knowledge co-production, a refocusing and extending of the initially formulated research questions, adapting them to local details and updating them as the local situation evolved. Subsequently, after a phase of disciplinary-based information gathering and analysis, the results were combined to provide a comprehensive picture of the situations, and the challenges and the options for problem solving were identified. The analysis was evaluated by scientific reviewers, and the options identified in a feedback-loop by local and regional stakeholders (Görg et al. 2014; Spangenberg et al. 2015b). The final results are co-produced knowledge and will be made available to all stakeholders in bilateral discussions or focus group meetings. They will finally be disseminated to the public at large via TV programs and (in Vietnam) a TV comedy show as a tested means of communicating ecological engineering to farmers (Heong et al. 2008). In LEGATO, scenario development was based on climate change and land use projections, derived from expert knowledge. Farmers and decision makers were asked regarding their expectations, but the answers given by the former were vague and by the latter, either summaries of the respective 5-year plan (Vietnam) or a bit like election campaign promises (the Philippines).

To facilitate scientific communication with stakeholders, a River Basin Information Centre (RBIC) was established in the project region in DaNangin during the last phase of the LUCCi project. At the centre, stakeholders could access to project results in the form of graphics, posters, reports, and brochures. All these materials were written in both Vietnamese and English language. RBIC offers a cross-sectoral neutral space to discuss fair water allocation and land management strategies and helps to improve the communication among the water and land use related stakeholders in the river basin. Additionally, the team of the LUCCi project also communicated the project results and data using a River Basin Information System.

Interdisciplinary communication among researchers, the basis for any TDR, was gradually intensified as the projects progressed. A basic approach to promote communication between the researchers in the respective projects was organizing regular project meetings or annual conferences for all researchers. This was usually preceded or followed by activities of field research where different teams could coordinate their different approaches. Regular interactions greatly contributed to shape a common understanding of objectives, terminologies, and methods beyond disciplinary boundaries (see also D.1 and D.2).

### Cutting across phase A and B

#### D. 1 Facilitate continuous formative evaluation

Each regional project took different measures to monitor and evaluate the respective TDR throughout the project period. So far, two assessments were conducted in SuMaRiO to evaluate interdisciplinary collaboration among German researchers: questionnaires and a SWOT (Strengths, Weaknesses, Opportunities, Threats) analysis. Such internal assessments provided inter alia a better understanding about the practice of interdisciplinary collaboration and how it could be improved (c.f. Podestá et al. 2013). In SURUMER, measures for monitoring and evaluation which was led by the monitoring and strategy team were developed. The measures included a joint problem analysis in the plenary and reflection of the objectives by all researchers after the first year, project meetings twice a year with subprojects reports to exchange information on activities and share preliminary results between subprojects (= monitoring), discussions on “integration issues” (modelling, scenarios, implementation activities), and publication of quarterly newsletters in which processes were reported and which were synthesized into annual reports. In LUCCi, the project progress and research results were presented and discussed each year in a consortium workshop which brought together all researchers and relevant stakeholders in addition to regular small project workshops organized by subproject research teams.

All regional projects were subject to a milestone evaluation by scientific reviewers commissioned by BMBF 1.5 years after the initiation of the project and a midterm evaluation about 3 years after project initiation. The evaluations were critical and helped to improve TDR (e.g., emphasizing the necessity to conduct a stakeholder analysis in all projects). However, as the review team consisted of reviewers of all projects, the level of knowledge regarding each project was very unevenly distributed, resulting in questions of varying relevance and quality. The value of advice of the reviewers depends strongly on the reviewers’ familiarity with the local situations.

#### D. 2 Mitigate conflict constellations

In all projects, conflict management was not a central issue and institutional processes foreseen for dealing with conflicts were not activated so far. However, measures were taken to support and enhance mutual understanding among researchers as well as between researchers and stakeholders. These measures focus on communication, information exchange, and trust building. For example, SuMaRiO PhD meetings were organized annually at different universities to provide a platform for interactions among doctoral students and post-doctoral researchers. In SURUMER, discourses were established to improve the internal communication regarding the importance of stakeholder communication. In LEGATO, a workshop was held to familiarize natural scientists with social science methods thus improving interdisciplinary understanding. To save experimental equipment from sabotage, natural scientists of the LEGATO project recognized the necessity of building trustful social relations with local famers (owners and workers) at experimental sites; therefore, farmers were informed and involved in setting up the experiments.

#### D. 3 Enhance capabilities for and interest in participation

Over the course of the project, researchers responsible for TDR made considerable efforts to enhance the capacities and interests of disciplinary researchers and stakeholders in participating in TDR. Stakeholder workshops in all projects were conducted in a way that allowed maximum participation and interactive discussions. Local languages were used (via translation when necessary) during interviews, workshops, and roundtable/focus group discussions to enable interview partners and participants to articulate their perspectives and to engage in meaningful deliberations. On the field, farmers were involved in the LEGATO project to set up the experiments. Citizen science which was implemented in the LEGATO project was a way for enhancing the capabilities of farmers to participate. Within the respective projects, regular exchanges among researchers were facilitated (see also B.2, D.1, and D.2) and joint focal points were set, for example the development of scenarios and integrated models as boundary objects. Additionally, joint publications were used as an incentive for enhancing interdisciplinary collaboration among researchers, for instance in SuMaRiO and LEGATO projects. Enhancing and sustaining the interest of stakeholders in participation were more challenging. In SuMaRiO, the decision support system has been used as an instrument to sustain the interest of the key stakeholders from the water sector in participation. To enhance the interests of stakeholders, SURUMER project team shifted the focus of stakeholder discussion to very practical farming issues (see also B.2). Providing research outputs is, therefore a way to enhance the interest of stakeholders who according to the SURUMER project did not want always to give information only.

## Lessons learned

Along the time span of the four evaluated projects, a number of outcomes have demonstrated the benefits of joint projects that integrate the expertise of different disciplinary backgrounds with stakeholder knowledge. Particularly, system understanding of researchers and stakeholders about the variety of issues could be improved and transformation pathways were discussed. By the time of this formative evaluation, system and goal knowledge have been integrated while the integration of transformation knowledge is ongoing. Experience during these years has shown that doing TDR is challenging. Due to the top-down governance and hierarchical institutional structures, the involvement of stakeholders from different sectors and levels is particularly difficult. The general guideline of stakeholder involvement and management provided by the “bridge-project” GLUES was useful, but it needed to be adapted to the specific conditions in the project areas. Researchers in charge of stakeholder involvement and management benefitted from the two-day workshop on stakeholder dialogues that was organized annually by the GLUES team. The workshops provided a platform for exchange and mutual learning among the 12 regional projects funded by BMBF.

In all projects there is a clear concept about TDR regarding stakeholder involvement and how stakeholder knowledge could be integrated with scientific knowledge. However, as TDR is still in its infancy, many researchers are not familiar yet with this research mode. This resulted in a lack of understanding and integration among disciplinary researchers in the first phase of the research projects. After internal workshops and some pressures exerted by external reviewers, the TDR approach, with some adjustments, could be implemented with better understanding and stronger support from researchers within the project.

The (early) involvement of the stakeholders is important to create project ownership and to motivate agents (stakeholders) to effectively take up project outcomes (Talwar et al. 2001). In all projects, however, it was unclear who the right discussion partners (i.e., stakeholder representatives who are knowledgeable and/or actually involved in decision making) were and who had the power to take decisions in the study areas at the beginning of the project, although key organizations were known. Stakeholder analyses were conducted as a result of the recommendation of the external reviewers to help identify relevant and important stakeholders by asking the question “who is in and why?” (Reed et al. 2009). To establish contacts with the right stakeholders and get them involved in transdisciplinary processes, it is certainly useful to have a project leader who has long term experience in the project area or better a project partner who is influential and has broad networks with stakeholders. In Asia, informal networks or “friendships” are often emphasized.

Trust building between stakeholders and researchers is essential in TDR. Trust enables stakeholders and researchers to engage in cooperative behaviour to address shared problems (Gray et al. 2012), including data sharing. In all projects, there was initially a feeling of distrust or at least suspicious unfamiliarity between stakeholders and researchers (either national or foreign), especially if the researchers were newcomers. During the first visit of the LEGATO team at the study sites, researchers could not access information about important cultural issues related to inheritance rules, gender, and the role of traditional knowledge due to the lack of trust. In SuMaRiO, governmental stakeholders were generally reluctant to provide data and information to foreign researchers, for example for hydrological modelling. While this may partially be due to lack of trust, staff of governmental organizations and scientists stated that access to certain data (e.g., daily streamflow data) was not possible due to legal restrictions. In some cases, data could be purchased, but it could be very expensive as experienced by the LUCCi project in Vietnam and by the LEGATO project in the Philippines. In another cases of the LEGATO project in Vietnam, data access could be facilitated through cooperation. This indicates that emphasizing mutual benefits can be a way to overcome data access problems. Furthermore, trusts can be built via informal networks.

In all regional projects, it was clear that good communication among researchers and particularly with stakeholders is important in TDR for knowledge integration. However, communication between foreign researchers and stakeholders in the four projects was hampered not only by language barriers but also by the culturally different ways of communication. The SuMaRiO team experienced that open discussions about certain issues, such as water quality and agricultural land expansion, were avoided as they were deemed to be “sensitive” and could be related to state secrets (van den Hoek et al. 2012). This was frustrating for foreign researchers used to open discussions. In a participatory setting where discussion is encouraged (e.g., at workshops), participants, especially those from lower hierarchical structure, were also frequently reserved and did not want to express their opinions in the presence of their superiors. This might be a result of negative experiences in the past (in China, for instance, the generation that actively experienced the cultural revolution seemed to be particularly cautious). Therefore, while some information given by stakeholders was inconsistent or contradictory, it remained unclear whether this was due to divergent knowledge or the reluctance of stakeholders to reveal available information. In LEGATO, it was common that farmers discussed lively among themselves before a joint answer to a question was given.

Language barriers are related to insufficient command of English as the international lingua franca or of the national language, also in cases where ethnic minorities do not speak it. Communication via translation (written or oral) was usually necessary in all projects, including the translation of key terms such as “ecosystem services” and “transdisciplinary research” (Spangenberg et al. 2014). The problem is that translating information from one language to another by translators can cause a loss in (important) information, especially when it is done orally. This may be caused by translators not being familiar with the research fields, but also occurs if the translator choose to not translate correctly what s/he regards as not appropriate to be translated, for political, cultural or for politeness reasons (Nord 2006). In situations experienced by the LEGATO team, some information was not revealed not due to a lack of trust of stakeholders in the foreign researchers but distrust in the “external translators” (i.e., professional translators from the capital city). Therefore, it is necessary to select translators who are appropriate for the specific project areas.

As mentioned before, strong support from all disciplinary researchers within the project consortium is essential for the achievement of the project goals. However, in all projects, researchers had different levels of interest in the overall TDR project. Most of the researchers in the project team wanted to focus on disciplinary research results (e.g., obtaining state-of-the-art measurements or improving disciplinary modelling methods), while only a few of them were open for interdisciplinary integration. For example, some doctoral students wanted to focus strongly on their own research projects because they needed to complete their theses within the planned timeframes. Delays in their projects due to difficulties in the project areas (e.g., no access to sampling sites or the lack of access to data for modelling) caused frustrations, which led to lower motivation and interest to commit to the overall transdisciplinary project. TDR suffers from the tension between obtaining academic merits and the delivery of policy-relevant outputs. This tension may become increasingly apparent as a TDR project evolves (Podestá et al. 2013). Measures can be taken to sustain the interest of researchers in TDR as demonstrated by different projects described here, including joint publications, workshops for interdisciplinary knowledge integration by e.g., scenario development or integrated modelling, and workshops for doctoral students involved in the TDR project. These measures have to be taken for promoting the willingness of researchers to adjust and integrate their disciplinary approaches.

## Conclusions

Four TDR projects in the field of sustainable land and water management have been conducted in China, Vietnam, and the Philippines. To improve the design of TDR projects in particular in those countries, we evaluated the four projects with respect to transdisciplinary knowledge integration and organization of TDR projects using the guiding questions developed by Lang et al. (2012). The results of the formative evaluation show that TDR did occur in all four projects but achieved different levels of stakeholder involvement as well as different degrees of integration of stakeholder knowledge. These variances can be explained by differences in the political conditions and as well as by the scale and comprehensiveness of the problem fields addressed in the individual projects. The difficulty of getting stakeholder involved to the degree desired in TDR projects is not exclusive for Asian countries. Similar problems were also encountered in other regions such as Africa and the USA as well as other case studies that deal with complex sustainability problems (Wiek et al. 2012, 2015). However, there are specific conditions in Asia.

Based on the experiences gained so far, we provide the following recommendations for the design and implementation of future TDR in Asia, especially in China, Vietnam, and the Philippines. Some recommendations may also be applicable in other contexts.

- The strong (in)formal and top-down hierarchies (including administrative position, social standing of a person, gender, and age, all of which are overlapping) in China and Southeast Asian countries are a barrier (or sometimes a benefit) to getting stakeholders involved in transdisciplinary processes. Under such conditions, people from as top as possible should be contacted, while those who are important informal multipliers at the lower hierarchical level also need to be sought for. Both are only possible through the established networks of the local project partners or the long term experience of the project leader. To sustain the relationships with the stakeholders, direct contact with local actors through extended periods or recurrent visits is also highly advisable.
- Stakeholder involvement in Asian countries is likely to require some financial compensation for the stakeholder representatives. Therefore, funding agencies need to provide some budget for financing stakeholder involvement beyond travel costs.
- Integration of stakeholder knowledge can be suboptimal when approaches and methods are used that are inappropriate for the local socio-cultural contexts. TDR approaches and methods need to be adapted to the local socio-cultural contexts and ways of communication, keeping in mind administrative, gender, age and other hierarchies.
- Effective, balanced, and trustworthy communication with stakeholders needs to be ensured in TDR, also for the benefit of data sharing. Supportive, competent, and trusted local project partners are required for facilitating communication as well as for building the network with relevant stakeholders. Local project partners should be engaged to communicate project results in native language and to obtain data required for research work, for example modelling.
- External translators not familiar to the stakeholders can be a potential source of distrust, even if trust in the foreign scientists is given. Within such cases, translators known to stakeholder representatives should be engaged unless local project partners can take the role as translators or foreign scientists can communicate in local languages. For high-level stakeholder workshops, it is recommended to employ professional translators familiar with the fields.
- Interdisciplinary collaboration forms the basis of TDR. Within TDR projects, specific measures are needed throughout the project duration to promote interdisciplinary collaboration beyond general project meetings. These measures include workshops for interdisciplinary knowledge integration (e.g., scenario development or integrated modelling) and joint publications but also regular communication of organizational issues.
- TDR processes aim at scientific results that are useful for solving real-world problems as well at social learning. Therefore, researchers need to explicitly elicit stakeholder perspectives (e.g., by actor modelling) and to listen carefully to what stakeholders express (in particularly Asian stakeholders may not express themselves in a direct way). They need to ensure that stakeholder perspectives are fully considered when identifying strategies. At the same time, they should make stakeholders aware that co-learning with researchers is also an important outcome of a TDR process.

It is important to emphasize that TDR is a recursive process. It requires intensive communication among researchers and stakeholders as well as continual adaptation and specific know-how. Sufficient financial resources over a long period of time (more than 5 years) should, therefore, be provided for TDR projects as well as for building capacity of researchers interested and involved in TDR. In addition, funding for a preparation phase is required to enable a joint problem identification and the definition of detailed research questions with stakeholders before the detailed proposal for the TDR project is written. Then, the positive impacts of TDR on sustainable development and (transdisciplinary) science can be further strengthened and sustained over a longer time period.

The described TDR processes led to the identification of some jointly developed management options. In LEGATO project, the establishment of flower strips in rice production landscapes as a means of biological pest control and the use of mass media campaigns for promoting sustainable rice production in Vietnam was agreed on (Westphal et al. 2015). In SURUMER, the need for training workshops on responsible pesticide usage was identified as a means for capacity building for local farmers. To facilitate scientific communication with stakeholders, the LUCCi project established a River Basin Information Centre (RBIC) in the project region. In the SuMaRiO project, severe political constraints prevented the joint identification of specific management options. While social learning has been observed in an anecdotal way during all TDR processes, conclusive evaluations of social learning have not (yet) been performed. We suggest that when planning TDR projects, an approach for identifying in what way project outcomes are related to the specific design of the TDR process should be developed. The methodological framework of Wiek et al. (2014), for example, further develops the Lang et al. (2012) approach towards the evaluation of tangible and intangible outcomes, by facilitating the evaluation of usable products, increase of knowledge and decision capacity, enhancement of networks, and transformational changes.
